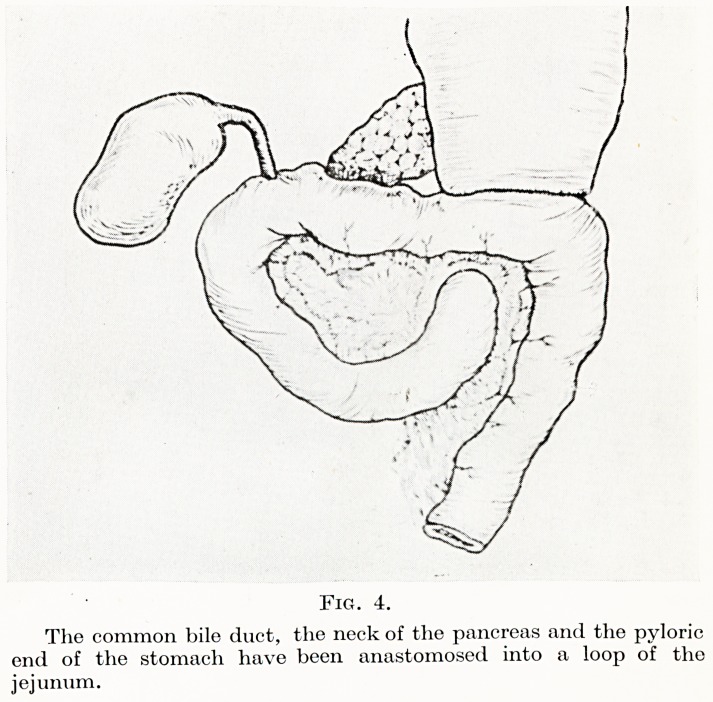# Resection of the Head of the Pancreas
*Notes of case shown at the Society's Clinical Meeting, December 11th, 1946.


**Published:** 1947

**Authors:** F. D. Murphy

**Affiliations:** Hon. Assistant Surgeon, Royal United Hospital, Bath. Consulting Surgeon, Trowbridge and Devizes District Hospitals


### RESECTION OF THE HEAD OF THE PANCREAS*

BY

F. D. MURPHY, O.B.E., F.R.C.S.

*Hon. Assistant Surgeon, Royal United Hospital, Bath.*
*Consulting Surgeon, Trowbridge and Devizes District Hospitals.*

---

RESECTION of the head of the pancreas for carcinoma has carried a high mortality, due both to absence of pancreatic secretions from the digestive tract, and to the formation of internal pancreatic fistulae. The aim of the modern operation is to re-establish communication between the pancreas and the intestine.

For the last four years several methods have been described by which this is achieved. They follow the same general principles, namely, radical excision of most or all of the duodenum and the head of the pancreas followed by anastomosis of stomach, pancreas and common duct or gall-bladder to the small intestine. The differences in detail warrant a short discussion.

1. *Excision of most* versus *all of duodenum.* Some of the methods advocate excision of the whole duodenum. That portion extending from the superior mesenteric artery to the duodeno-jejunal flexure is very difficult of access and its removal is hazardous and adds considerably to the length of the operation. Division of the duodenum just proximal to the artery is a much simpler procedure and does not make the operation less radical as the remaining portion can be considered surgically remote from the site of growth and in no way makes the excision of the whole of the head of the pancreas more difficult.

2. *Order of anastomosis.* Cholangitis is a not infrequent remote complication from which deaths have been reported. Gastro-jejunal anastomosis proximal to that of the gall-bladder or bile-duct in my opinion adds to this risk, and it is desirable when possible that the former should be the more distal. Cattell countered this by performing an entero-anastomosis proximal and distal to the choledocho-jejunostomy, but this does not get over a second objection to a proximal gastro-jejunal anastomosis, the danger of stomal ulcer.

3. *Cholecyst-jejunostomy* versus *Choledocho-jejunostomy.* This problem does not arise in the two-stage operation in which there is no alternative to cholecyst-jejunostomy. The objection to choledocho-jejunostomy is that it is more difficult and takes longer to perform. However, in these cases the diameter of the common duct may be anything up to one inch, which makes for ease of anastomosis. If cholecyst-jejunostomy is decided upon, then simple ligation of the common duct is not enough, as it may lead to leakage of bile and

* Notes of case shown at the Society's Clinical Meeting, December 11th, 1946.

### PLATE I

Fig. 1.

The hepatic flexure has been pushed downwards off the duodenum, the greater and lesser curvatures of the stomach near the pylorus freed and the duodenum reflected to the left exposing the vena cava and the common bile duct.

A. Common bile duct. B. Hepatic artery.

Fig. 2.

The stomach has been cut across and the superior mesenteric vessels freed and the portal vein dissected away from the pancreas.

A. Superior pancreatico-duodenal vein.
B. Inferior pancreatico-duodenal vessels.

### PLATE II

Fig. 3.

The pyloric fragment of the stomach and the duodenum with the whole of the head of the pancreas have been removed and the distal end of the duodenum closed.

A. Common bile duct.
B. Cut end of superior pancreatico-duodenal vein.
C. Cut end of inferior pancreatico-duodenal vein.

Fig. 4.

The common bile duct, the neck of the pancreas and the pyloric end of the stomach have been anastomosed into a loop of the jejunum.

should be reinforced by inversion of the stump by a purse-string suture. Both procedures certainly take longer than a choledocho-jejunostomy. The greatest objection to choledocho-jejunostomy is perhaps that cholangitis is more likely to occur.

In the case here reported, the method I set out to use is that described by Professor C. A. Pannett in the *British Journal of Surgery*, July, 1946, to whom I am indebted for his kind permission to use the diagrams reproduced below. However, owing to extensive chronic pancreatitis around the growth in the head of the pancreas, I had to modify the procedure at some points.

The first step after opening the abdomen and assessing operability is to expose the third part of the duodenum by dissecting off the hepatic flexure of the colon and pushing it and the proximal part of the transverse colon downwards. When this has been completed the stomach is transected between clamps about 1½–2 inches above the pylorus. The posterior peritoneum just lateral to the second part of the duodenum is divided, and the duodenum and the head of the pancreas dissected medially off the inferior vena cava and the common duct divided about half an inch from its termination. Next, the uncinate process of the pancreas is dissected from between the superior mesenteric vein and the inferior vena cava. The pancreas is now divided through its neck, in the course of which the gastro-duodenal artery and the superior and inferior pancreatico-duodenal veins are ligatured. The duodenum is now cleared just proximal to where it passes under the superior mesenteric vessels, divided between clamps and the distal end closed with two layers of sutures. The first part of the jejunum is then taken up in front of the transverse colon and the common bile-duct, neck of pancreas and stomach anastomosed to it. Drainage is established through a one-inch subcostal wound just lateral to the right rectus muscle. This is preferable to drainage through the lower end of the wound, which is almost certain to break down if there is much loss of pancreatic fluid.

*Case Report.* C.S. Male. Aged 44. Admitted to medical unit of the Royal United Hospital, Bath, on 6.7.46, suffering from jaundice of six weeks' duration which investigation proved to be obstructive in type.

Operation 29.7.46. Obstruction found to be due to carcinoma of the head of the pancreas. Cholecyst-gastrostomy performed.

Postoperative progress: Transient fading of jaundice with the appearance of bile pigments in stools. At the end of three weeks the jaundice was as intense as before operation and the stools again clay-coloured. Second operation advised.

Operation 29.8.46. Cholecyst-gastrostomy undone and openings in gall-bladder and stomach oversewn. (Failure of the anastomisis was found to be due to herniation of gastric mucous membrane through a small stoma.) Resection of the head of the pancreas carried out as described above. The operation did not present unusual difficulties except at two points, namely, the dissection of the uncinate process and the clearing of the portal and superior mesenteric veins. The latter are very thin-walled vessels, and in spite of taking the utmost care were torn in two places. Repair was carried out with continuous fine catgut suture. Postoperative progress was satisfactory for the first two days. On the third day the patient developed collapse of the left lower lobe and the amount of drainage suddenly increased. This caused a rapid deterioration and his condition remained critical for a week. The fluid loss from the drainage tube on the fifth day amounted to nearly three pints and contained bile and pancreatic juices. This was countered by intravenous glucose saline and plasma. September 15th. Jaundice decreasing : Bile-pigments in stools : Discharge from fistula abating. September 24th. Discharge much less in amount : Bile still present but no evidence of pancreatic ferments. October 4th. Discharge ceased : Appetite good : Getting up. February, 1947. Patient's condition excellent : back to normal weight. Examination of fæces shows that fat and protein digestion is proceeding normally but that there is a slight deficiency in carbohydrate digestion.

## Figures and Tables

**Fig. 1. f1:**
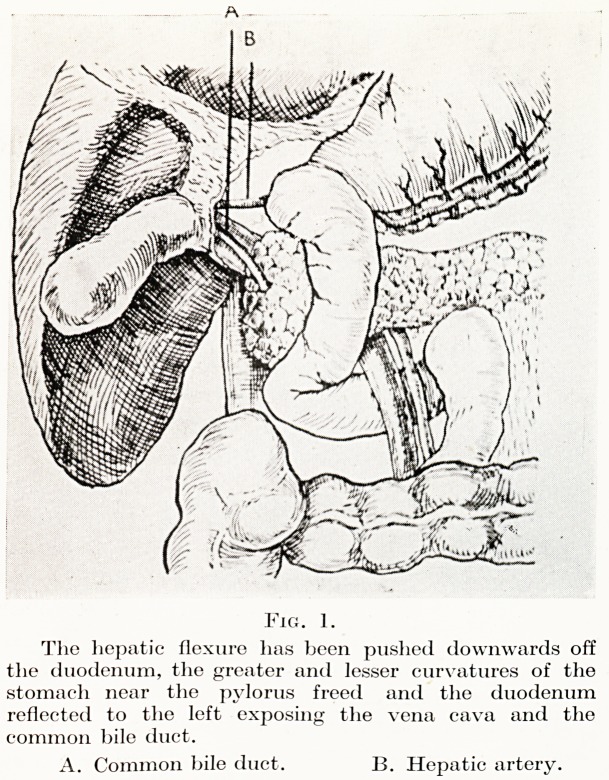


**Fig. 2. f2:**
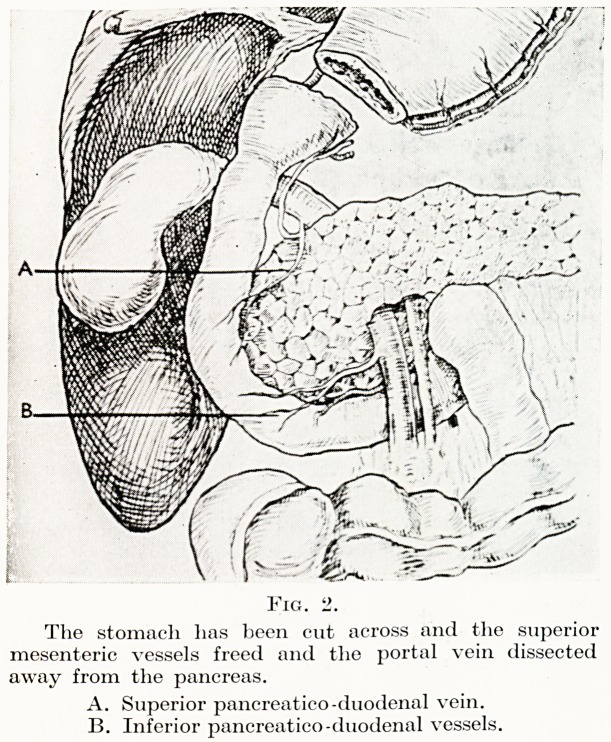


**Fig. 3. f3:**
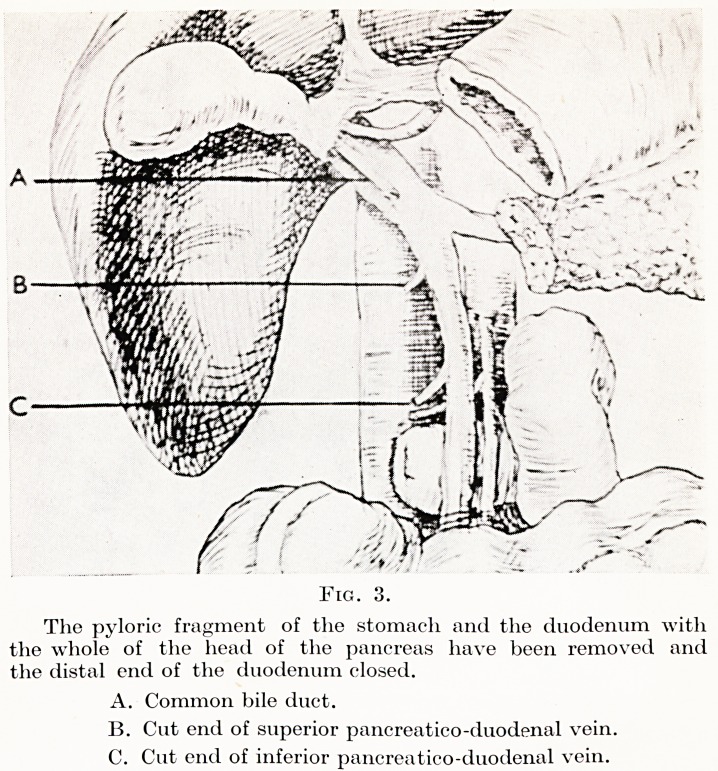


**Fig. 4. f4:**